# Novel associations of UDP-glucuronosyltransferase 2B gene variants with prostate cancer risk in a multiethnic study

**DOI:** 10.1186/1471-2407-13-556

**Published:** 2013-11-22

**Authors:** Adriana C Vidal, Cocoa Tucker, Joellen M Schildkraut, Ricardo M Richardson, Megan McPhail, Stephen J Freedland, Cathrine Hoyo, Delores J Grant

**Affiliations:** 1 Department of Obstetrics and Gynecology, and Program of Cancer Detection, Prevention and Control, Duke University School of Medicine, Durham, NC 27710, USA; 2 Department of Biology and Cancer Research Program, JLC-Biomedical/Biotechnology Research Institute, North Carolina Central University, Durham, NC 27707, USA; 3 Department of Community and Family Medicine and Program of Cancer Detection, Prevention and Control, Duke University Medical Center, Durham, NC 27705, USA; 4 Department of Surgery, Division of Urology, Duke University Medical Center, Durham, NC, 27710, USA; 5 Department of Surgery, Durham VA Medical Center, Durham, NC 27705, USA; 6 Duke Prostate Center, Duke University Medical Center, Durham, NC 27710, USA; 7 Department of Pathology, Duke University Medical Center, Durham, NC 27710, USA

## Abstract

**Background:**

We have previously shown that a functional polymorphism of the *UGT2B15* gene (rs1902023) was associated with increased risk of prostate cancer (PC). Novel functional polymorphisms of the *UGT2B17* and *UGT2B15* genes have been recently characterized by *in vitro* assays but have not been evaluated in epidemiologic studies.

**Methods:**

Fifteen functional SNPs of the *UGT2B17* and *UGT2B15* genes, including *cis*-acting *UGT2B* gene SNPs, were genotyped in African American and Caucasian men (233 PC cases and 342 controls). Regression models were used to analyze the association between SNPs and PC risk.

**Results:**

After adjusting for race, age and BMI, we found that six *UGT2B15* SNPs (rs4148269, rs3100, rs9994887, rs13112099, rs7686914 and rs7696472) were associated with an increased risk of PC in log-additive models (p < 0.05). A SNP *cis*-acting on *UGT2B17* and *UGT2B15* expression (rs17147338) was also associated with increased risk of prostate cancer (OR = 1.65, 95% CI = 1.00-2.70); while a stronger association among men with high Gleason sum was observed for SNPs rs4148269 and rs3100.

**Conclusions:**

Although small sample size limits inference, we report novel associations between *UGT2B15* and *UGT2B17* variants and PC risk. These associations with PC risk in men with high Gleason sum, more frequently found in African American men, support the relevance of genetic differences in the androgen metabolism pathway, which could explain, in part, the high incidence of PC among African American men. Larger studies are required.

## Background

Prostate cancer is the second leading cause of cancer-related deaths in men, after lung cancer [1]. The incidence of prostate cancer has increased over the past twenty years and African American men have been disproportionally affected compared to other racial/ethnic groups [2-7]. In the U.S., the incidence of prostate cancer among African Americans is more than 60% higher than in Caucasians, and the mortality rate in African Americans is twice that of Caucasian men [8,9]. Although differences in incidence and mortality rates may be due, in part, to race/ethnicity, socioeconomic conditions and availability of health care [10], familial aggregation studies suggest that genetic factors may also be contributing to prostate cancer demographic disparity. Candidate gene approaches involving hormone metabolic pathways have been examined in prostate cancer association studies, however results from these studies have not been replicated [11,12]. Nonetheless, current therapies are primarily targeted at specific androgen biosynthetic pathways [13], thus, improved knowledge on genetic variants associated with both androgen metabolism and prostate cancer risk is important.

The *UDP-glucuronosyltransferase* (*UGT*) genes code for enzymes that convert a diverse group of xenobiotic and endobiotic substances into lipophilic compounds, facilitating clearance from the body as part of the phase II liver detoxification system [14]. These enzymes are subdivided into families according to similarities in amino acid sequence and target substrates dictated by the specificity of their amino-terminal ends [15]. A specific subfamily, UGT2B, includes two enzymes, UGT2B17 and UGT2B15, which are also expressed extrahepatically in the basal and luminal epithelium of the prostate, respectively. These enzymes exhibit specificity for androgen metabolites such as testosterone, dihydrotestosterone (DHT), androsterone (ADT), and androstane-3α,17β-diol (3β-diol) in prostate tissue and cell lines [16,17].

Functional polymorphisms of the *UGT2B* gene family such as the copy number variant (CNV) of *UGT2B17*, and the aspartic acid (D or nucleotide G) to tyrosine substitution (Y or nucleotide T) found in codon 85 of the *UGT2B15* gene (*UGT2B15*^*D85Y*^, rs1902023) have been identified [18,19]. Studies suggest that men with 0 copies of the *UGT2B17* gene are unable to break down testosterone through the UGT2B pathway and subsequently secrete negligible amounts of urinary testosterone compared to men with at least one copy of *UGT2B17*[18,20]. Experimental evidence showed that the minor allele (T) of the *UGT2B15* variant, *UGT2B15*^*D85Y*^, causes the enzyme to have an increased V_max_ activity when compared to the presence of the major allele (G) [19]. Subsequently, the resulting phenotype of this *UGT2B15* polymorphism is a quicker androgen metabolite clearance which may raise the “effective” amount of steroids within the prostate and decrease risk for prostate cancer [21]. These two major *UGT2B* variants (the CNV in *UGT2B17* and the polymorphism in *UGT2B15)* have been evaluated in relation to prostate cancer risk, with inconsistent findings [22-32]. Discrepancies could have been due to the genetic heterogeneity of the populations studied, as well as variable sample sizes of these populations. We have recently shown that individuals with a major allele (G) of the *UGT2B15*^*D85Y*^ polymorphism (rs1902023) have higher risk of prostate cancer when compared to individuals homozygous for the “rapid clearance” minor allele (Y) [33]. In the same study, the *UGT2B17* CNV showed no association with prostate cancer risk [33]. Recently, an additional 7 novel *UGT2B15* SNPs that are in strong linkage disequilibrium (LD) with the *UGT2B15*^*D85Y*^gene variant, have been identified by re-sequencing the promoter and exon one regions of the *UGT2B15* gene, using DNA samples from Yoruba (YRI), CEPH/European (CEU), and Japanese/Chinese (ASN) populations [34]. Most of these *UGT2B* variants have not been evaluated in relation to prostate cancer risk in population-based studies or in studies that included African American men. In this present work, we examined associations between functional SNPs of *UGT2B17*, *UGT2B15* and three other related *UGT2B* SNPs, and prostate cancer risk among African American and Caucasian men.

## Methods

### Study population

The details of participant accrual for this case control study have been previously reported [35]. In brief, male subjects from the Durham Veterans Affairs Medical Center (DVAMC) in Durham, North Carolina, who were undergoing a prostate needle biopsy between January 2007 and October 2011, were consecutively contacted in a hospital-based, case control study. Eligibility criteria for cases included age >18 years, undergoing a prostate biopsy for concerns of potential prostate cancer after presentation with elevated PSA and/or abnormal digital rectal examination, and prostate cancer positive classification by pathological review of biopsy tissue. Of the 759 men with a biopsy indication who were screened for eligibility, 539 (759/539 = 71% participation rate) provided written consent to participate. Twenty two men elected not to follow through and of the 517 men that underwent a biopsy, 233 had a biopsy with histological evidence of prostate cancer. Controls were recruited from the DVAMC Internal Medicine Clinic; eligibility criteria were age >18 and having a PSA test conducted at the DVAMC within the same time frame, but not recommended to undergo biopsy. Of the 768 men who met eligibility criteria for controls, 377 provided written consent (768/377 = 49% participation rate). Questionnaires were administered to prostate cancer cases prior to biopsy and to controls to assess risk factors including race and age. Institutional Review Board approval was obtained at North Carolina Central University, Duke University, and the DVAMC and all patients signed an informed consent prior to enrollment.

### Genotyping

*UGT2B* variants were selected for genotyping from previous published reports as well as dbSNP and SNP Tags from the Genome Variation Server (GVS) (Table 1). Eight were from the *UGT2B15 gene,* 4 were from the *UGT2B17* gene and 3 were *cis-*acting on the *UGT2B15* and/or *UGT2B17* gene expression. *UGT2B15* SNPs that were genotyped for this study include rs1580083, rs1960733 (formerly rs34050522), rs9994887 (formerly rs35513228), rs13112099, rs7866914 (formerly rs34027331), rs7696472, rs4148296, rs3100 and rs17221777. *UGT2B15* SNPs rs1580083, rs1960733 (formerly rs34050522), rs9994887 (formerly rs35513228), rs13112099, rs7686914 (formerly rs34027331) and rs7696472 were identified from the re-sequencing of the *UGT2B15* promoter and exon 1 in 56 HapMap samples from YRI, CEU, and East Asian (ASN) populations [34]. Other *UGT2B15* SNPs genotyped, rs4148296 (T523K) and rs3100 (3′UTR) were characterized by differential allelic expression assays and re-sequencing, respectively [36]. The final *UGT2B15* SNP, rs17221777, was identified in a previous study [18]. *UGT2B17* SNPs that were genotyped for this study SNPs include rs72551386, rs59678213, rs7435827, rs7686008, rs7671342, rs35994121, and rs35140421. *UGT2B17* SNPs rs7435827, rs7686008, rs7671342, rs35994121, and rs35140421 were identified as *UGT2B17* Tag SNPs in the GVS and dbSNP databases. *UGT2B17* SNP rs59678213 was identified as a novel polymorphism in a transcriptional binding site in the promoter region of *UGT2B17*[37]. Four SNPs were genotyped in this study, rs6822259, rs17147338, rs2168047 and rs4557343, that were identified in a SNP discovery study and characterized as cis-acting on *UGT2B17* (rs6822259), *UGT2B15* (rs4557343), and *UGT2B17* and *UGT2B15* (rs17147338, rs2168047) gene expression [38]. Of the twenty SNPs, five were excluded from analysis because they were either monoallelic (rs72551386, rs17221777, rs3599412, and rs3514942) in the study population or the genotyping assay failed (rs455734).

**Table 1:** ***UGT2B15 *** **and *UGT2B17 *** **genotyped SNPs and Minor alleles**

**SNP**	**Function class**	**Minor allele/MAF**			**Reference**
		**All**	**Afr. American**	**Caucasian**	
***UGT2B15 SNPs***					
rs1580083	Promoter	T/0.74	0.70	0.80	34
rs1960773	Promoter (Nrf2)	T/0.73	0.70	0.76	34
rs9994887	Promoter	A/0.73	0.67	0.78	34
rs13112099	Promoter	T/0.74	0.70	0.78	34
rs7686914	Promoter	T/0.73	0.68	0.78	34
rs7696472	Promoter	G/0.73	0.68	0.76	34
rs4148269	Nonsyn (T523K)	C/0.70	0.70	0.85	36
rs3100	3′UTR	T/0.72	0.53	0.85	36
***UGT2B17 SNPs***					
rs59678213	Promoter (FoxA1)	C/0.32	0.24	0.67	37
rs7435827	Intron	G/0.60	0.30	0.76	GVS
rs7686008	Intron	T/0.18	0.34	0.06	GVS
rs7671342	Intron	G/0.41	0.41	0.41	GVS
***Cis acting on UGT2B expression***					
rs6822259**^**	Intergenic	C/0.32	0.30	0.30	38
rs17147338**^^**	Intergenic	T/0.16	0.30	0.079	38
rs2168047**^^**	Distal promoter	A/0.67	0.73	0.63	38

**^**cis-acting on *UGT2B17* expression (36).

**^^**cis-acting on *UGT2B17* and *UGT2B15* expression (36).

GVS – genome variation server.

DNA was isolated from peripheral blood by standard DNA isolation (Qiagen Inc., Valencia, CA, U.S.A.) and quantified by ultraviolet spectrophotometry. Prior to genotyping, DNA concentration was determined using PicoGreen assay (Life Technologies, Gaithersburg, MD) and measured using the fluorescence intensity measurements plotted against a standard curve that was generated from the average fluorescence intensity of standards run in replicate. Based on the PicoGreen quantification, 10 ng of genomic DNA from each sample was used in the iPlex assay for Sequenom-iPlex Genotyping (Sequenom Inc., San Diego, CA). The Sequenom Mass Array (Sequenom Inc., San Diego, CA) was used and the assays for all SNPs were designed by Sequenom online assay tools (Assay Designer 4.0) at the David H. Murdock Research Institute (DHMRI) Genomics Laboratory (Kannapolis, NC). The data were analyzed by Sequenom-Typer 4.0. The Sequenom-iPlex genotyping and analysis was validated with CEPH gDNA controls when performing the iPlex assay and scanning on the MALDI-TOF Mass Spectrometer. At the time of this analysis, 585 samples were submitted and successfully produced good spectra for genotyping at a failure rate of <2.2% for the majority of SNPs. The Post-QC (Call Rate) of SNPs rs59678213 and rs7435827 was 92.7% and 94.7%, respectively. The assays included DHMRI control DNA (CEPH) on each plate in duplicate that were checked for concordance for each SNP. Sample duplicates were individually inspected for genotype consistency. Genotypes from duplicate samples were 100% concordant.

### Statistical analysis

We examined whether genetic variants of *UGT2B17* and *UGT2B15* genes were associated with prostate cancer risk and whether these associations varied by race and prostate cancer grade. Low grade prostate cancer was defined as Gleason sum <8 and high-grade prostate cancer was defined as Gleason ≥ 8. Potential confounders (age, height, and BMI) were normally distributed overall and were treated as continuous variables in modeling. Race was self-reported and categorized as African American, Caucasian and other (Native American and Latino). Family history of prostate cancer included maternal lineage (maternal uncle) or paternal lineage (father, brother or paternal uncle) (yes or no) and these combined. Data on tobacco use at the time of enrollment was also self-reported as yes or no.

Descriptive statistics (means, SD), and percentages for cases and controls were estimated using *X*^2^ tests for categorical variables, and Wilcoxon rank-sum test for continuous variables. Unconditional logistic regression was used to estimate the odd ratios (ORs) and 95% confidence intervals (95% CI) for the association between genotypes and prostate cancer risk. Multinomial logistic regression models were used to explore whether associations between genotypes and prostate cancer risk varied by grade of prostate cancer at diagnosis, using controls as reference. Confounders adjusted for in all models were age, race, and BMI. All analyses were done using SAS version 9.3 (SAS Institute, Inc., Cary, NC).

## Results

Table 2 describes the clinical characteristics of the study participants. Prostate cancer cases (n = 233) and controls (n = 342) were comparable in height (p = 0.31), however controls (mean BMI = 30.77, SD = 6.05) had slightly higher BMI than cases (mean BMI = 29.25, SD = 5.60, p = 0.002) and were slighter younger than cases (p = 0.005). African American men comprised 63% of the cases and 41% of the controls (p <0.0001). A family history of prostate cancer was reported by 14% of cases and 10% of the controls. (p = 0.16). Most cases and controls reported tobacco use (p = 0.43). Ninety-one percent of the cases had low-grade prostate cancer (Gleason sum < 8) vs. 9% of cases with high grade prostate cancer (Gleason sum ≥ 8).

**Table 2:** Risk factor characteristics for prostate cancer participants

**Risk factors**	**Cases n = 233 n (%)**	**Controls n = 342 n (%)**	**p-value**
**Age - mean (SD)**	63.34 (6.37)	61.32 (7.49)	p = 0.005
**Height (in.)**	69.65 (2.92)	69.91 (3.03)	p = 0.31
**Obesity (BMI)**	29.25 (5.60)	30.77 (6.05)	p = 0.002
**Race**			p = <.0001
**African American**	146 (62.66)	140 (40.93)	
**Caucasian**	87 (37.34)	202 (59.06)	
**Family history PC**			p = 0.16
**Yes**	32 (13.73)	34 (9.94)	
**No**	201 (86.26)	308 (90.05)	
**Tobacco use**			p = 0.43
**Yes**	225 (96.56)	334 (97.66)	
**No**	8 (3.43)	8 (2.33)	
**Gleason sum <8 (134**	211 (91.34)		
**Black/77 White)**		NA	
**≥8 (10 Black/10 White)**	20 (8.66)		

The functional variants of the *UGT2B15* and *UGT2B17* SNPs were assessed for associations with prostate cancer risk and summarized in Table 3. After adjusting for age, race and BMI, four *UGT2B15* SNPs, rs9994887, rs13112099, rs7686914 and rs7696472, were associated with increased risk of prostate cancer, when compared to the minor allele (Log additive model OR = 1.49, 95% CI = 1.09, 2.04; OR = 1.48, 95% CI = 1.08, 2.03, OR = 1.48, 95% CI = 1.08, 2.03, OR = 1.49, 95% CI = 1.06, 2.00, respectively). *UGT2B15* SNPs rs4148269 and rs3100, were also associated with increased risk for prostate cancer (Log additive model OR = 1.45, 95% CI = 1.03, 2.03; OR = 1.46, 95% CI = 1.08, 1.98, respectively). These data suggest that these associations were allele dose-dependent (Table 3). None of the *UGT2B17* SNPs studied were associated with prostate cancer risk, however the *UGT2B17 cis*-acting SNP, rs17147338, showed borderline significance for increased risk of prostate cancer in an allele dose dependent fashion (OR = 1.65, 95% CI = 1.00, 2.70), after adjusting for age, race and BMI (Table 3). SNPs in high LD with *UGT2B15*^*D85Y*^*,* rs9994887, rs13112099, rs7686914, and rs796472 [34] were associated with increased risk of prostate cancer in an allele dose-dependent manner for both high-grade (OR = 2.34, 95% CI = 1.22, 4.49; OR = 2.31, 95% CI = 1.20, 4.44; OR = 2.31, 95% CI = 1.20, 4.44; OR = 2.26, 95% CI = 1.18, 4.33, respectively) and low-grade prostate cancer lesions (OR = 1.94, 95% CI = 1.06, 3.53; OR = 1.93, 95% CI = 1.06, 3.52; OR = 1.94, 95% CI = 1.06, 3.53; OR = 1.99, 95% CI = 1.10, 3.63, respectively) after adjusting for age, race, and BMI (Table 4). Intriguingly, SNPs rs4148269 and rs3100 were associated with increased PC risk in high grade lesions only (OR = 1.96, 95% CI = 1.01, 3.82; OR = 2.16, 95% CI = 1.18, 3.95).

**Table 3:** *ORs for the associations between *UGT2B15 * **and *UGT2B17 *** **SNPs and PC**

**SNP # Allele**	**Cases n (%)**	**Controls n (%)**	OR (95% CI)*
**UGT2B15**			
**rs1580083**			
TT	2 (0.86)	5 (1.47)	Reference
TA	150 (64.38)	249 (73.45)	2.32 (0.23-23.81)
AA	81 (34.76)	85 (25.07)	3.24 (0.31-33.93)
AA/TA	231 (99.14)	334 (98.52)	2.54 (0.25-26.00)
**Log additive**			1.43 (0.91-2.26)
**rs1960773**			
TT	1 (0.43)	5 (1.47)	Reference
GT	144 (62.61)	244 (71.98)	--
GG	85 (36.96)	90 (26.55)	--
TT/GT	229 (99.56)	334 (98.52)	--
**Log additive**			--
**rs9994887**			
AA	38 (16.31)	91 (26.84)	Reference
AG	108 (46.35)	158 (46.61)	1.94 (1.06-3.53)
GG	87 (37.34)	90 (26.55)	2.38 (1.24-4.55)
GG/AG	195 (83.69)	248 (73.15)	2.10 (1.18-3.71)
**Log additive**			1.49 (1.09-2.04)
**rs13112099**			
TT	38 (16.31)	91 (26.61)	Reference
GT	110 (47.21)	163 (47.66)	1.93 (1.06-3.52)
GG	85 (36.48)	88 (25.73)	2.35 (1.22-4.51)
GG/GT	195 (83.69)	251 (73.39)	2.07 (1.12-3.67)
**Log additive**			1.48 (1.08-2.03)
**rs7686914**			
TT	38 (16.45)	91 (26.84)	Reference
CT	109 (47.19)	160 (47.20)	1.94 (1.06-3.55)
CC	84 (36.36)	88 (25.96)	2.34 (1.22-4.50)
CC/CT	193 (83.55)	248 (73.81)	2.10 (1.17-3.70)
**Log additive**			1.48 (1.08-2.03)
**rs7696472**			
GG	38 (16.31)	92 (26.97)	Reference
AG	110 (47.21)	157 (46.04)	2.00 (1.10-3.63)
AA	85 (36.48)	90 (26.39)	2.30 (1.20-4.40)
AA/AG	195 (83.69)	249 (73.02)	2.10 (1.18-3.72)
**Log additive**			1.49 (1.06-2.00)
**rs4148269**			
CC	43 (18.45)	79 (23.23)	Reference
CA	72 (30.90)	160 (47.05)	0.81 (0.45-1.46)
AA	115 (49.35)	101 (29.70)	1.98 (1.02-3.85)
AA/CA	190 (81.54)	261 (76.76)	1.10 (0.63-1.90)
**Log additive**			1.45 (1.03-2.03)
**rs3100**			
TT	54 (23.78)	106 (31.08)	Reference
TC	68 (29.95)	140 (41.05)	1.04 (0.60-1.81)
CC	105 (46.25)	95 (27.85)	2.18 (1.19-3.99)
CC/TC	173 (76.21)	235 (68.91)	1.41 (0.86-2.31)
**Log additive**			1.46 (1.08-1.98)
**Cis acting on UGT2B expression**			
**rs2168047**			
AA	53 (22.84)	59 (17.45)	Reference
GA	112 (48.27)	169 (50.00)	0.86 (0.48-1.53)
GG	67 (28.87)	110 (32.54)	1.00 (0.52-1.88)
GG/GA	179 (77.15)	279 (82.54)	0.90 (0.52-1.57)
**Log additive**			1.01 (0.74-1.38)
**rs6822259**			
CC	3 (1.29)	12 (3.52)	Reference
CT	58 (25.00)	97 (28.44)	1.48 (0.36-6.02)
TT	171 (73.70)	232 (68.03)	1.34 (0.34-5.31)
TT/CT	229 (98.70)	329 (96.48)	1.39 (0.35-5.43)
**Log additive**			1.02 (0.68-1.53)
**rs17147338**			
CC	168 (72.1)	285 (83.58)	Reference
CT	55 (23.61)	51 (14.96)	1.68 (0.96-2.97)
TT	10 (4.29)	5 (1.47)	2.41 (0.42-13.94)
TT/CT	65 (27.9)	56 (16.42)	1.73 (1.00-3.00)
**Log additive**			1.65 (1.00-2.70)
**UGT2B17**			
**rs7435827**			
GG	53 (24.20)	106 (32.61)	Reference
GA	43 (19.63)	78 (24.00)	0.67 (0.35-1.30)
AA	123 (56.16)	141 (43.38)	0.90 (0.49-1.66)
AA/GA	166 (75.79)	219 (67.38)	0.80 (0.46-1.38)
**Log additive**			0.97 (0.71-1.36)
**rs7686008**			
AA	168 (71.79)	281 (82.4)	Reference
AT	34 (14.53)	32 (9.38)	1.40 (0.69-2.79)
TT	32 (13.68)	28 (8.21)	1.40 (0.66-2.96)
TT/AT	66 (28.21)	60 (17.6)	1.39 (0.79-2.44)
**Log additive**			1.21 (0.85-1.73)
**rs7671342**			
GG	37 (15.94)	56 (16.37)	Reference
GA	46 (19.83)	85 (24.85)	0.86 (0.43-1.73)
AA	149 (64.22)	201 (58.77)	1.32 (0.73-2.40)
AA/GA	195 (84.05)	286 (83.62)	1.17 (0.65-2.08)
**Log additive**			1.21 (0.91-1.61)
**rs59678213**			
CC	42 (19.44)	81 (25.63)	Reference
TC	46 (21.29)	74 (23.42)	0.84 (0.43-1.63)
TT	128 (59.26)	161 (50.94)	0.85 (0.45-1.59)
TT/TC	174 (80.55)	235 (74.37)	0.84 (0.47-1.50)
**Log additive**			0.92 (0.68-1.26)

*Adjusted for age, race and BMI.

**Table 4:** *ORs for the associations between *UGT2B15 * **and *UGT2B17 *** **SNPs and PC in low grade and high grade PC cases**

**SNP # Allele**	**Low gleason <8 n (%)**	**Adjusted OR (95% CI)**	**High gleason ≥8 n (%)**	Adjusted OR* (95% CI)
**UGT2B15**					
**rs1580083**					
TT	0 (0)	Reference	2 (10.00)	Reference	
TA	139 (65.88)	--	10 (50.00)	0.20 (0.02-2.42)	
AA	72 (34.12)	--	8 (40.00)	0.33 (0.02-4.36)	
AA/TA	211 (100.0)	--	18 (90.00)	0.13 (0.02-0.74)	
**Log additive**		--		3.20 (0.30-33.45)	
**rs1960773**					
TT	0 (0)	Reference	1 (5.26)	Reference	
GT	132 (63.16)	--	11 (57.89)	--	
GG	77 (36.84)	--	7 (36.84)	--	
GG/GT	209 (100.0)	--	18 (94.73)	--	
**Log additive**		--		--	
**rs9994887**					
AA	32 (15.17)	Reference	5 (25.00)	Reference	
AG	101 (47.87)	2.13 (1.13-4.00)	7 (35.00)	0.86 (0.19-3.80)	
GG	78 (36.97)	2.53 (1.30-4.99)	8 (40.00)	1.27 (0.26-6.17)	
GG/AG	179 (84.83)	2.27 (1.24-4.14)	15 (75.00)	1.00 (0.25-3.90)	
**Log additive**		1.94 (1.06-3.53)		2.34 (1.22-4.49)	
**rs13112099**					
TT	32 (15.16)	Reference	5 (25.00)	Reference	
GT	103 (48.81)	2.12 (1.13-3.98)	7 (35.00)	0.84 (0.19-3.71)	
GG	76 (36.01)	2.50 (1.26-4.93)	8 (40.00)	1.30 (0.26-6.28)	
GG/GT	179 (84.83)	2.25 (1.23-4.10)	15 (75.00)	1.00 (0.25-3.87)	
**Log additive**		1.93 (1.06-3.52)		2.31 (1.20-4.44)	
**rs7686914**					
TT	32 (15.23)	Reference	7 (36.83)	Reference	
CT	102 (48.57)	2.14 (1.14-4.02)	7 (36.84)	0.86 (0.19-3.78)	
CC	76 (36.19)	2.50 (1.26-4.92)	5 (26.32)	1.30 (0.26-6.30)	
CC/CT	178 (84.76)	2.25 (1.23-4.13)	12 (63.16)	1.00 (0.26-3.92)	
**Log additive**		1.94 (1.06-3.54)		2.31 (1.20-4.44)	
**rs7696472**					
AA	32 (15.17)	Reference	8 (40.00)	Reference	
AG	103 (48.82)	2.20 (1.16-4.12)	7 (35.00)	0.86 (0.19-3.82)	
GG	76 (36.02)	2.43 (1.23-4.81)	5 (25.00)	1.26 (0.26-6.11)	
GG/AG	179 (84.83)	2.30 (1.25-4.16)	12 (60.00)	1.00 (0.26-3.92)	
**Log additive**		1.99 (1.10-3.63)		2.26 (1.18-4.33)	
**rs4148269**					
CC	38 (18.18)	Reference	5 (26.32)	Reference	
CA	66 (31.58)	0.76 (0.42-1.40)	5 (26.32)	2.16 (0.23-20.26)	
AA	105 (50.24)	1.70 (0.86-3.36)	9 (47.36)	13.55 (1.34-136.99)	
AA/CA	171 (81.81)	1.00 (0.56-1.74)	14 (73.68)	3.94 (0.47-2.58)	
**Log additive**		0.81 (0.45-1.47)		1.96 (1.01-3.82)	
**rs3100**					
TT	46 (22.33)	Reference	7 (36.84)	Reference	
TC	64 (31.07)	1.04 (0.60-1.83)	4 (21.05)	1.10 (0.17-6.86)	
CC	96 (46.60)	1.96 (1.06-3.63)	8 (42.10)	8.22 (1.31-51.60)	
CC/TC	160 (77.66)	1.34 (0.81-2.22)	12 (63.16)	2.42 (0.50-11.83)	
**Log additive**		1.04 (0.60-1.81)		2.16 (1.18-3.95)	
**rs2168047**					
AA	49 (23.22)	Reference	3 (15.79)	Reference	
GA	99 (46.92)	0.78 (0.43-1.42)	12 (63.16)	2.61 (0.30-22.38)	
GG	63 (29.86)	0.96 (0.50-1.84)	4 (21.05)	1.92 (0.18-20.07)	
GG/GA	162 (76.77)	0.84 (0.48-1.48)	16 (84.21)	2.38 (0.30-19.86)	
**Log additive**		0.85 (0.51-1.40)		1.01 (0.53-1.91)	
**rs6822259**					
CC	3 (1.43)	Reference	0 (0)	Reference	
CT	50 (23.81)	1.25 (0.30-5.10)	7 (35.00)	--	
TT	157 (74.76)	1.27 (0.32-5.03)	13 (65.00)	--	
TT/CT	207 (98.57)	1.26 (0.32-4.95)	20 (100.00)	--	
**Log additive**		1.11 (0.68-1.79)		--	
**rs17147338**					
CC	154 (72.99)	Reference	12 (60.00)	Reference	
CT	48 (22.75)	1.64 (0.92-2.93)	7 (35.00)	2.78 (0.65-11.96)	
TT	9 (4.27)	1.92 (0.30-12.18)	1 (5.00)	12.86 (0.91-181.95)	
TT/CT	57 (27.01)	1.67 (0.94-2.91)	8 (40.00)	3.41 (0.89-13.08)	
**Log additive**		1.70 (1.00-3.01)		2.44 (0.42-12.17)	
**UGT2B17**					
**rs7435827**					
GG	50 (25.00)	Reference	3 (16.67)	Reference	
GA	38 (19.00)	0.67 (0.34-1.31)	4 (22.22)	0.73 (0.11-4.72)	
AA	112 (56.00)	0.87 (0.46-1.63)	11 (61.11)	1.33 (0.26-6.84)	
AA/GA	150 (75.00)	0.78 (0.44-1.37)	15 (83.33)	1.03 (0.23-4.65)	
**Log additive**		0.67 (0.35-1.32)		0.90 (0.49-1.66)	
**rs7686008**					
AA	154 (72.99)	Reference	12 (60.00)	Reference	
AT	29 (13.74)	1.25 (0.61-2.58)	4 (20.00)	5.42 (1.05-27.96)	
TT	28 (13.27)	1.30 (0.60-2.80)	4 (20.00)	5.05 (0.72-35.66)	
TT/AT	57 (27.01)	1.27 (0.71-2.28)	8 (40.00)	5.30 (1.19-23.55)	
**Log additive**		1.41 (0.70-2.84)		1.42 (0.67-3.01)	
**rs7671342**					
GG	34 (16.19)	Reference	3 (14.28)	Reference	
GA	43 (20.48)	0.89 (0.44-1.81)	4 (19.05)	0.61 (0.08-4.60)	
AA	133 (63.33)	1.31 (0.71-2.41)	14 (66.67)	1.28 (0.26-6.35)	
AA/GA	176 (83.81)	1.17 (0.64-2.11)	18 (85.71)	1.05 (0.22-5.04)	
**Log additive**		0.86 (0.43-1.73)		1.30 (0.72-2.36)	
**rs59678213**					
CC	40 (20.20)	Reference	2 (11.76)	Reference	
TC	41 (20.71)	0.99 (0.54-1.81)	4 (23.53)	2.77 (0.27-28.17)	
TT	117 (59.09)	0.76 (0.40-1.45)	11 (64.71)	3.60 (0.38-33.75)	
TT/TC	158 (79.79)	0.76 (0.42-1.38)	15 (88.23)	3.21 (0.37-27.47)	
**Log additive**		1.02 (0.56-1.82)		1.18 (0.63-2.21)	

*Adjusted for age, race and BMI.

Restricting analyses by race/ethnicity, and after adjusting for age and BMI, further revealed that these associations were present in African American men (rs9994887, OR = 1.66, 95% CI = 1.05, 2.63; rs13112099, OR = 1.66, 95% CI = 1.03, 2.63; rs7686914, OR = 1.64, 95% CI = 1.03, 2.63; rs7696472, OR = 1.64, 95% CI = 1.03, 2.63; rs4148269, OR = 2.04, 95% CI = 1.19, 3.44; and rs3100, OR = 1.89, 95% CI = 1.22, 2.94) (Table 5). However, the interaction terms for these SNPs and race were not statistically significant (p > 0.4) for all six SNPs. For both groups, carrying the *UGT2B17* or *cis*-acting SNPs was not associated with prostate cancer risk (Table 5).

**Table 5:** *ORs for the associations between *UGT2B15 * **and *UGT2B17 *** **SNPs and PC in African American and Caucasian men**

	**African American**			**Caucasian**		
**SNP # Allele**	**Cases n (%)**	**Controls n (%)**	Adjusted OR (95% CI)*	**Cases n (%)**	**Controls n (%)**	Adjusted OR (95% CI)*
**UGT2B15**							
**rs1580083**							
TT	0 (0)	1 (0.72)	Reference	2 (2.30)	4 (20.9)	Reference	
TA	86 (58.90)	94 (68.12)	---	64 (73.56)	155 (77.11)	1.22 (0.10-14.43)	
AA	60 (41.10)	43 (31.16)	---	21 (24.14)	42 (1.99)	1.36 (0.11-16.96)	
AA/TA	146 (100.0)	137 (99.27)	---	85 (97.70)	159 (79.1)	1.25 (0.11-14.73)	
**Log additive**			--			1.12 (0.58-2.17)	
**rs1960773**							
TT	0 (0)	2 (1.45)	Reference	1 (1.16)	3 (23.38)	Reference	
GT	82 (56.94)	93 (67.39)	--	62 (72.09)	151 (75.12)	--	
GG	62 (43.06)	43 (31.16)	-	23 (26.74)	47 (1.49)	--	
GG/GT	144 (100.0)	136 (98.55)	--	85 (98.83)	198 (76.62)	--	
**Log additive**			--			--	
**rs9994887**							
AA	16 (10.96)	28 (20.44)	Reference	22 (25.29)	63 (31.50)	Reference	
AG	65 (44.52)	63 (45.99)	1.92 (0.72-5.10)	43 (49.43)	93 (46.50)	1.93 (0.90-4.14)	
GG	65 (44.52)	46 (33.58)	2.96 (1.09-8.04)	22 (25.29)	44 (22.00)	1.87 (0.77-4.54)	
GG/AG	130 (89.04)	109 (79.56)	2.34 (0.93-5.88)	65 (74.71)	137 (68.50)	1.91 (0.92-3.96)	
**Log additive**			1.66 (1.05-2.63)			1.35 (0.88-2.08)	
**rs13112099**							
TT	16 (10.96)	28 (20.00)	Reference	22 (25.29)	63 (31.18)	Reference	
GT	67 (45.89)	68 (48.57)	1.96 (0.74-5.20)	43 (49.43)	95 (47.03)	1.88 (0.88-4.05)	
GG	63 (43.15)	44 (31.43)	2.94 (1.07-8.05)	22 (25.29)	44 (21.78)	1.87 (0.77-4.54)	
GG/GT	130 (89.04)	112 (80.00)	2.34 (0.93-5.88)	65 (74.71)	139 (68.81)	1.88 (0.91-3.91)	
**Log additive**			1.66 (1.03-2.63)			1.35 (0.88-2.08)	
**rs7686914**							
TT	16 (11.03)	28 (20.14)	Reference	22 (25.58)	63 (31.50)	Reference	
CT	67 (46.21)	67 (48.20)	2.00 (0.75-5.31)	42 (48.84)	93 (46.50)	1.88 (0.87-4.05)	
CC	62 (42.75)	44 (31.65)	2.94 (1.07-8.05)	22 (25.58)	44 (22.00)	1.87 (0.77-4.54)	
CC/CT	129 (88.96)	111 (79.86)	2.37 (0.94-5.96)	64 (74.42)	137 (68.50)	1.88 (0.91-3.91)	
**Log additive**			1.64 (1.03-2.63)			1.35 (0.88-2.08	
**rs7696472**							
GG	16 (10.96)	29 (20.70)	Reference	22 (25.29)	63 (31.66)	Reference	
AG	67 (45.89)	67 (47.86)	1.96 (0.74-5.20)	43 (49.43)	90 (45.23)	2.01 (0.93-4.32)	
AA	63 (43.15)	44 (31.43)	2.94 (1.07-8.05)	22 (25.29)	46 (23.12)	1.77 (0.73-4.27)	
AA/AG	130 (89.04)	111 (79.28)	2.34 (0.93-5.88)	65 (74.71)	136 (68.34)	1.93 (0.93-4.04)	
**Log additive**			1.64 (1.03-2.63)			1.31 (0.86-2.00)	
**rs4148269**							
CC	9 (6.25)	11 (7.86)	Reference	34 (39.53)	68 (34.00)	Reference	
CA	34 (23.61)	58 (41.43)	0.73 (0.20-2.70)	38 (44.19)	102 (51.00)	0.89 (0.46-1.73)	
AA	101 (70.14)	71 (50.71)	2.34 (0.67-7.83)	14 (16.28)	30 (15.00)	1.46 (0.57-3.77)	
AA/CA	135 (93.75)	129 (92.14)	1.65 (0.51-5.34)	52 (60.46)	132 (66.00)	0.99 (0.53-1.85)	
**Log additive**			2.04 (1.19-3.44)			1.12 (0.71-1.75)	
**rs3100**							
TT	18 (12.86)	32 (23.02)	Reference	36 (41.38)	74 (36.63)	Reference	
TC	31 (22.14)	42 (30.22)	1.57 (0.54-4.55)	37 (42.53)	98 (48.51)	0.91 (0.47-1.72)	
CC	91 (65.00)	65 (46.76)	3.36 (1.36-8.30)	14 (16.09)	30 (14.85)	1.48 (0.58-3.78)	
CC/TC	122 (87.14)	107 (76.97)	2.75 (1.15-6.58)	51 (58.62)	128 (63.36)	1.00 (0.54-1.84)	
**Log additive**			1.89 (1.22-2.94)			1.12 (0.72-1.75)	
**Cis acting on UGT2B expression**							
**rs2168047**							
AA	42 (28.97)	36 (26.09)	Reference	11 (12.64)	23 (11.50)	Reference	
GA	69 (47.59)	65 (47.10)	0.83 (0.39-1.75)	43 (49.43)	104 (52.00)	0.92 (0.35-2.42)	
GG	34 (23.44)	37 (26.81)	0.91 (0.37-2.21)	33 (37.94)	73 (36.50)	1.07 (0.39-2.93)	
GG/GA	103 (71.03)	102 (73.91)	0.85 (0.42-1.72)	76 (87.35)	177 (88.50)	0.98 (0.38-2.48)	
**Log additive**			0.94 (0.61-1.47)			1.07 (0.67-1.72)	
**rs6822259**							
CC	1 (0.69)	4 (2.86)	Reference	2 (2.30)	8 (3.98)	Reference	
CT	25 (17.24)	38 (27.14)	0.61 (0.03-10.56)	33 (37.93)	59 (29.35)	1.60 (0.86-2.96)	
TT	119 (82.07)	98 (70.00)	0.91 (0.05-14.99)	52 (59.77)	134 (66.67)	0.70 (0.14-3.54)	
TT/CT	144 (99.31)	136 (97.14)	0.83(0.05-13.61)	85 (97.70)	193 (96.01)	1.47 (0.81-2.67)	
**Log additive**			1.34 (0.68-2.77)			1.23 (0.75-2.03)	
**rs17147338**							
TT	91 (62.33)	100 (71.43)	Reference	77 (88.51)	0 (0)	Reference	
CT	46 (31.51)	35 (25.00)	1.72 (0.84-3.54)	9 (10.34)	16 (7.96)	--	
CC	9 (6.16)	5 (3.57)	1.51 (0.23-9.88)	1 (1.15)	185 (92.04)	--	
CC/CT	55 (37.67)	40 (28.57)	1.70 (0.85-3.40)	10 (11.49)	16 (7.96)	--	
**Log additive**			1.53 (0.84-2.82)			--	
**UGT2B17**							
**rs7435827**							
GG	13 (9.35)	13 (9.56)	Reference	40 (50.00)	93 (49.21)	Reference	
GA	22 (15.83)	27 (19.85)	0.47 (0.12-1.92)	21 (26.25)	51 (26.98)	0.78 (0.37-1.66)	
AA	104 (74.82)	96 (70.59)	0.80 (0.24-2.68)	19 (23.75)	45 (23.81)	0.86 (0.40-1.85)	
AA/GA	126 (90.65)	123 (90.44)	0.72 (0.22-2.41)	40 (50.00)	96 (50.79)	0.82 (0.44-1.52)	
**Log additive**			1.08 (0.64-1.85)			1.08 (0.75-1.59)	
**rs7686008**							
AA	83 (56.85)	92 (66.19)	Reference	84 (96.55)	189 (93.56)	Reference	
AT	32 (21.92)	25 (17.99)	1.65 (0.74-3.69)	2 (2.3)	7 (3.47)	0.70 (0.13-3.71)	
TT	31 (21.23)	22 (15.83)	1.51 (0.67-3.44)	1 (1.15)	6 (2.97)	0.99 (0.10-10.04)	
TT/AT	63 (43.15)	47 (33.81)	1.58 (0.83-3.01)	3 (3.45)	13 (6.44)	0.78 ( 0.20-3.08)	
**Log additive**			1.28 (0.86-1.90)			0.88 (0.33-2.36)	
**rs7671342**							
GG	22 (15.07)	23 (16.43)	Reference	15 (17.44)	33 (16.34)	Reference	
GA	25 (17.12)	35 (25.00)	0.66 (0.24-1.88)	21 (24.42)	50 (24.75)	1.06 (0.42-2.67)	
AA	99 (67.81)	82 (58.57)	1.51 (0.63-3.64)	50 (58.14)	119 (58.91)	1.15 (0.51-2.59)	
AA/GA	124 (84.93)	117 (83.57)	1.21 (0.52-2.84)	71 (82.55)	169 (83.66)	1.12 (0.51-2.46)	
**Log additive**			1.36 (0.90-2.08)			1.07 (0.73-1.59)	
**rs59678213**							
CC	10 (7.35)	8 (5.97)	Reference	32 (40.00)	73 (40.11)	Reference	
TC	19 (13.97)	24 (17.91)	0.45 (0.10-2.00)	27 (33.75)	50 (27.47)	1.04 (0.49-2.20)	
TT	107 (78.68)	102 (76.12)	0.65 (0.18-2.41)	21 (26.25)	59 (32.42)	0.80 (0.37-1.71)	
TT/TC	126 (92.65)	126 (94.02)	0.62 (0.17-2.24)	48 (60.00)	109 (59.89)	0.91 (0.48-1.73)	
**Log additive**			0.99 (0.64-1.69)			1.11 (0.76-1.61)	

*Adjusted for age and BMI.

To investigate the extent to which these SNPs were associated in our subjects we calculated correlation coefficients among the controls as shown in Table 6. The r^2^ values for the *UGT2B15* variants rs1580083, rs1960773, rs13112099, rs7686914, rs7696472 and rs9994887 ranged from 0.62 to 0.99/1.00 (p < 0.0001). *UGT2B15* variants rs4148269 and rs3100 were also strongly correlated (r^2^ = 0.85, p < 0.0001). Including both SNPs into the same regression model to evaluate whether one SNP would have a stronger association with PC risk did not yield additional insights. Notably, these two SNPs showed no correlation with the other SNPs that were highly associated with each other. *UGT2B17* SNP rs7435827 was moderately correlated with *UGT2B15* SNP rs2168047 (r^2^ = 0.36, p < 0.0001) and *UGT2B17* SNP rs7671342 (r^2^ = 0.32, p < 0.0001), and strongly correlated with *UGT2B17* SNP rs59678213 (r^2^ = 0.83, p < 0.0001); however these SNPs showed no association with prostate cancer risk. *UGT2B17* SNP rs59678213 was inversely associated with *UGT2B17* SNPs rs2168047 (r^2^ = −0.47, p < 0.0001), rs7686008 (r^2^ = −0.29, p < 0.0001) and rs7671342 (r^2^ = −0.49, p < 0.0001). No correlations were observed for the cis-acting *UGT2B* SNPs that were identified through the recent *UGT2B* SNPs discovery study [38]. All SNPs that showed associations with prostate cancer risk were in Hardy-Weinberg equilibrium in control samples, except for *UGT2B15* SNP rs3100.

**Table 6:** **Spearman correlation coefficients and p values for *UGT2B15 *** **and *UGT2B17 *** **SNPs**

	**rs1580083**	**rs1960073**	**rs9994887**	**rs13112099**	**rs7686914**	**rs7696472**	**rs4148269**	**rs3100**	**rs2168047**	**rs6822259**	**rs17147338**	**rs7435827**	**rs7686008**	**rs7671342**
rs1580083	1.000	0.894	0.625	0.637	0.638	0.625	0.002	0.000	0.000	0.000	0.002	0.001	0.000	0.002
		<.0001	<.0001	<.0001	<.0001	<.0001	0.458	0.780	0.749	0.873	0.366	0.527	0.720	0.414
rs1960073	0.894	1.000	0.631	0.650	0.645	0.631	0.001	0.000	0.001	0.001	0.002	0.000	0.000	0.003
	<.0001		<.0001	<.0001	<.0001	<.0001	0.669	0.931	0.634	0.614	0.366	0.946	0.686	0.284
rs9994887	0.625	0.631	1.000	0.989	0.989	0.973	0.000	0.000	0.002	0.000	0.003	0.010	0.001	0.006
	<.0001	<.0001		<.0001	<.0001	<.0001	0.685	0.776	0.384	0.894	0.296	0.069	0.549	0.145
rs13112099	0.637	0.650	0.989	1.000	1.000	0.984	0.001	0.070	0.002	0.000	0.004	0.009	0.001	0.005
	<.0001	<.0001	<.0001		<.0001	<.0001	0.033	0.627	0.461	0.970	0.263	0.093	0.510	0.176
rs7686914	0.638	0.645	0.989	1.000	1.000	0.984	0.001	0.001	0.002	0.000	0.004	0.009	0.001	0.005
	<.0001	<.0001	<.0001	<.0001		<.0001	0.033	0.627	0.462	0.970	0.264	0.094	0.510	0.177
rs7696472	0.625	0.631	0.973	0.984	0.984	1.000	0.002	0.002	0.001	0.000	0.003	0.007	0.001	0.006
	<.0001	<.0001	<.0001	<.0001	<.0001		0.044	0.443	0.523	0.961	0.282	0.139	0.619	0.161
rs4148269	0.002	0.001	0.000	0.001	0.001	0.002	1.000	0.849	0.000	0.000	0.007	0.046	0.059	0.021
	0.458	0.669	0.685	0.550	0.550	0.4217		<.0001	0.854	0.753	0.122	0.0001	<.0001	0.008
rs3100	0.000	0.000	0.000	0.070	0.001	0.002	0.849	1.000	0.003	0.001	0.002	0.018	0.021	0.015
	0.780	0.931	0.776	0.627	0.627	0.443	<.0001		0.332	0.530	0.362	0.016	0.007	0.023
rs2168047	0.000	0.001	0.002	0.002	0.002	0.001	0.000	0.003	1.000	0.007	0.001	0.356	0.043	0.139
	0.749	0.634	0.384	0.461	0.462	0.523	0.854	0.332		0.135	0.549	<.0001	0.0001	<.0001
rs6822259	0.000	0.001	0.000	0.000	0.000	0.000	0.000	0.001	0.007	1.000	0.023	0.004	0.006	0.000
	0.873	0.614	0.894	0.970	0.970	0.961	0.753	0.530	0.135		0.005	0.279	0.170	0.825
rs17147338	0.002	0.002	0.003	0.004	0.004	0.003	0.007	0.002	0.001	0.023	1.000	0.005	0.000	0.000
	0.366	0.366	0.295	0.263	0.264	0.282	0.122	0.362	0.549	0.005		0.223	0.895	0.873
rs7435827	0.001	0.000	0.010	0.009	0.009	0.007	0.046	0.018	0.356	0.004	0.005	1.000	0.162	0.318
	0.527	0.946	0.069	0.093	0.093	0.139	0.0001	0.016	<.0001	0.279	0.223		<.0001	<.0001
rs7686008	0.000	0.000	0.001	0.001	0.001	0.001	0.059	0.021	0.043	0.006	0.000	0.162	1.000	0.047
	0.720	0.686	0.549	0.510	0.510	0.619	<.0001	0.007	0.0001	0.170	0.895	<.0001		<.0001
rs7671342	0.002	0.003	0.006	0.005	0.005	0.006	0.021	0.015	0.139	0.000	0.000	0.318	0.047	1.000
	0.414	0.284	0.145	0.176	0.177	0.161	0.008	0.023	<.0001	0.825	0.873	<.0001	<.0001	
rs59678213	0.032	−0.001	0.078	0.071	0.070	0.058	0.116	0.045	−0.473	0.086	−0.077	0.830	−0.494	1.000
	0.577	0.985	0.170	0.211	0.213	0.303	0.034	0.42	<0.0001	0.217	0.168	<0.0001	<0.001	

## Discussion and conclusion

In this study we examined associations between functional SNPs of the *UDP-glucuronosyltransferase 2B* (*UGT2B*) genes and prostate cancer risk. After adjusting for age and BMI, we found that two *UGT2B15* SNPs, rs4148269 and rs3100, were associated with a two-fold increase risk of prostate cancer and the association was more apparent in African American men. In addition, SNPs rs9994887, rs13112099, rs7686914 and rs7696472 were also associated with increased risk of prostate cancer. These associations persisted either in low or high-grade prostate cancer lesions for SNPs rs9994887, rs13112099, rs7686914, and rs7696472. However, SNPs rs4148269 and rs3100 were only associated with increased risk of prostate cancer in high-grade prostate cancer lesions. These findings are novel and support the hypothesis that these SNPs may affect UDP-glucuronosyltransferase enzyme function, presumably by increasing the efficiency of androgen metabolite clearance compared to enzyme products of the *UGT2B* wild type genotype, as has been shown for *UGT2B15* SNP rs1902023 [21].

Associations between functional SNPs of the *UGT2B15* and *UGT2B17* genes and prostate cancer risk have been previously examined and have rendered conflicting results [22-32]. Discrepancies could be due in part to racial/ethnic differences in the populations studied but also to sample size and allele dosage. Comparison of two studies with different sample sizes revealed contradictory results: no associations between SNP *UGT2B15*^*D85Y*^ and prostate cancer risk were found in a study with a large sample size [9], whereas a study with a smaller sample size found an increased risk of prostate cancer associated with the *UGT2B15*^*D85Y*^ variant in Caucasian subjects [32]. In reference to allele dosage, we previously concluded [33], concordant with previous reports [31,32,39], that homozygous carriers of the dominant *UGT2B15*^*D85Y*^ (G) allele missense polymorphism, (rs1902023), had an almost three-fold higher risk of prostate cancer. Our current results further confirm that associations with *UGT2B15* polymorphisms are allele dose-dependent and may also be dependent on race/ethnicity. Furthermore, our study includes a large and multiethnic sample size in comparison to previous reports.

Some of our stratified analyses revealed that *UGT2B15* wild type alleles were significantly associated with increased risk of prostate cancer in African American men, but not in Caucasian men, suggesting that the wild type genotype may confer protection against prostate cancer in Caucasian men. While these findings require replication in larger studies, we provide new evidence that wild type genotypes for the *UGT2B15* gene more frequently found in African American men may increase the risk of prostate cancer, and thus may contribute to the present racial/ethnical disparities seen in prostate cancer incidence. Few studies have assessed the association of *UGT2B* genes with prostate cancer risk in African Americans. Park et al. [25] were one of the first to report that associations between *UGT2B17* polymorphisms and increased risk of prostate cancer were linked to race/ethnicity; they showed that Caucasian men carrying a *UGT2B17* deletion polymorphism had a two-fold increased risk of prostate cancer, but not Black men who carry the same deletion, compared to non-carriers [25]. Subsequent work supported these findings [22,26], which were further confirmed by others [23,24]. Our results show no significant associations of *UGT2B17* polymorphisms with prostate cancer risk in neither Caucasian nor African American men. Our findings that *UGT2B17* SNP rs59678213 was not associated with prostate cancer risk are consistent with those of others [22,24].

Four of the *UGT2B15* SNPs that conferred prostate cancer risk in this study are among seven SNPs (rs1580083, rs1960773, rs9994887, rs13112099, rs7686914, rs7696472, and rs1120265 [not in study]) that are in high linkage disequilibrium (LD) with *UGT2B15*^*D85Y*^ SNP rs1902023 [34]. The phenotype of the variations, established with reporter gene assays in the liver cancer cell line HepG2, showed that transcriptional activity was higher for constructs containing the major alleles when compared to constructs containing the minor alleles. When a construct with the minor allele of rs9994887 was transfected into a prostate cancer cell line, LNCaP, transcriptional activity was decreased by approximately 14%, compared to constructs containing the major allele [34], which suggested that rs9994887 may influence gene expression in the prostate [34]. Further experiments would have to be done to determine the impact of these variants on the kinetics and efficacy of UGT2B enzymes. Interestingly, the other three SNPs that showed associations with increased prostate cancer risk, rs13112099, rs7686914, and rs7696472 were highly correlated with rs9994887 (r^2^ = 0.99, 0.99, and 0.97, respectively). In addition, the three SNPs are all located upstream of rs9994887 (−1139 bp relative to translation start) while the other two SNPs are located downstream. While the impact of these variants on UGT2B15 enzyme kinetics has not been reported, the associations suggest that the phenotype of the enzyme may be similar to that of the *UGT2B15*^*85Y*^ variant or rs1902023 [19]. SNPs rs4148269 and rs3100 also demonstrated strong associations with decreased risk of PC, but did not show any correlation with the promoter SNPs in this study population. SNP rs4148269 has been characterized as a T523K amino acid substitution [40], however a comprehensive analysis of the enzyme phenotype has not been reported. This suggests that rs3100, which is highly correlated (r^2^ = 0.85) with rs4148269, may be important in the etiology of PC. Results from reporter gene assays showed that the major allele of rs3100 is associated with significantly higher gene expression in LNCaP cells when compared to constructs made with the minor allele [36].

The lack of association observed for the *UGT2B17* SNPs and PC risk was unexpected, as previous reports suggested that the UGT2B17 enzyme plays a major role in testosterone glucuronidation [20]. However results from a recent report comparing UGT2B17 and UGT2B15 mRNA and protein expression, suggest that *UGT2B15* may be negatively regulated in naïve and castration-resistant tumors while undetectable in lymph node metastases [41]. More *in vitro* analyses are required to understand the functional impact of these genetic variations. Nonetheless this is the first time these SNPs are reported in relation to prostate cancer risk.

The main limitation of this study is the sample size which limited our ability to simultaneously examine race/ethnicity in the associations with SNPs and prostate cancer grade. The low numbers of Caucasians may have limited our ability to detect significant associations within this group given that they were less likely to present a high grade PC tumor. The small sample size could also have been a contributing factor in the lack of significance for the cross-product term for the SNPs and race, however race/ethnicity was self-reported and we did not measure ancestral markers, which could indicate the percentage of African American ancestry of each subject. Also, we cannot exclude the possibility of potential confounding due to differences in MAFs between races. Nonetheless our study was conducted at the DVAMC which provides medical care for all former military personnel. This type of setting has important advantages. The Veterans Affairs Medical Centers (VAMC) are considered an equal-access health care settings that may control for health care availability among various U.S. ethnicities [42]. Moreover, the VAMCs provide a setting for PC case–control studies as this is the leading cancer reported in Veterans Affairs Central Registry (VACCR) [43].

Despite these limitations, in the current case–control study of African American and Caucasian men, we observed elevated risk of prostate cancer in African American men who carry the wild type of the functional *UGT2B15* gene variants. Future studies with larger sample sizes are needed to confirm these findings.

## Competing interests

No potential conflict of interest were disclosed.

## Authors’ contributions

DJG conceived and designed the experiments. MM and SJF provided the samples. ACV, CT, DJG and CH analyzed and interpreted the data. SJF, RMR and JMS contributed in the writing of the manuscript. ACV, CT, DJG and CH wrote the manuscript. All authors have read and approved the final manuscript.

## Pre-publication history

The pre-publication history for this paper can be accessed here:

http://www.biomedcentral.com/1471-2407/13/556/prepub
